# First report of glyphosate-resistant downy brome (*Bromus tectorum* L.) in Canada

**DOI:** 10.1038/s41598-022-21942-6

**Published:** 2022-11-07

**Authors:** Charles M. Geddes, Mattea M. Pittman

**Affiliations:** Agriculture and Agri-Food Canada, Lethbridge Research and Development Centre, 5403 1st Avenue South, Lethbridge, AB T1J 4B1 Canada

**Keywords:** Ecology, Plant sciences

## Abstract

Glyphosate is the most used herbicide worldwide, and is an important source of economical weed control in glyphosate-resistant crops, and conservation tillage systems, among other uses. Downy brome (*Bromus tectorum* L.), otherwise known as cheatgrass, is a highly invasive winter-annual grass weed in cropping systems, pastureland, and naturalized or ruderal areas in western North America. In 2021, a downy brome population remained uncontrolled following four applications of glyphosate in a glyphosate-resistant canola (*Brassica napus* L.) field located in Taber County, Alberta, Canada. All individuals from the subsequent generation of the population survived glyphosate treatment at the typical field rate (900 g ae ha^−1^). In dose-response bioassays, the putative glyphosate-resistant population exhibited 10.6- to 11.9-fold, 7.7- to 8.7-fold, 7.8- to 8.8-fold, and 8.3- to 9.5-fold resistance to glyphosate based on plant survival, visible control, and biomass fresh weight and dry weight, respectively, compared with two susceptible populations 21 days after treatment. Estimated glyphosate rates for 80% control of this population ranged from 2795 to 4511 g ae ha^−1^; well above common usage rates. This downy brome population represents the first glyphosate-resistant grass weed confirmed in Canada, and the second weed species exhibiting glyphosate resistance in the Canadian prairie region.

## Introduction

Glyphosate [Herbicide Resistance Action Committee (HRAC) Group 9] is the most used herbicide worldwide, and is an important source of economical weed control in glyphosate-resistant crops, and conservation tillage systems^1^. Glyphosate has been touted as a “once-in-a-century” herbicide due to its favorable properties including broad-spectrum and systemic activity on a wide range of plant species, low residual activity in soil, low mammalian toxicity, minimal environmental impact, low cost, and what was initially suggested to be low selection pressure for resistance in weeds^1–4^. However, 54 weed species have evolved resistance to glyphosate in recent decades, spanning 30 countries worldwide^5^.

The risk of selection for glyphosate-resistant weeds in the prairie region of Canada has increased in the past two decades largely due to the adoption of glyphosate-resistant crops {canola (*Brassica napus* L.), corn (*Zea mays* L.), soybean [*Glycine max* (L.) Merr.], and sugar beet (*Beta vulgaris* L.)} beginning in 1996^6^ and also reliance on glyphosate for cost-effective non-selective pre-plant weed control in conservation tillage systems^7^. In Alberta, glyphosate sales tripled between 1998 and 2018^8^, undoubtedly resulting in greater selection pressure for glyphosate-resistant weeds. In 2020, crops with genetically engineered glyphosate resistance were grown on about 3.1 million ha or 9.7% of the arable land in the prairie region of Canada (2,192,000 ha of canola, 518,000 ha of soybean, 350,000 ha of corn, and 14,000 ha of sugar beet)^9–11^. In this same region, about 86.6% of arable land is farmed using conservation tillage systems (direct-seeding or retaining most of the crop residue on the soil surface)^12^, most of which receives a pre-plant burndown application of glyphosate (either alone or mixed with other herbicide sites of action) annually. Repetitive use of glyphosate during multiple application windows in cropping systems [pre-plant, post-emergence (in glyphosate-resistant crops), pre-harvest, and post-harvest], and for targeted plant management in rangeland, naturalized, and ruderal areas, risks further evolution or spread of glyphosate-resistant weeds^4^.

Downy brome (*Bromus tectorum* L.), otherwise known as cheatgrass, is a winter-annual grass weed that was introduced to North America from Europe prior to 1861^13^. Reports suggest multiple isolated introductions of downy brome to North America due to its potential as a short-season “100-day” grass used for livestock feed^14,15^. Since then, it spread throughout most of the continent resulting in significant infestations in cropland, pastureland and ruderal areas in the semi-arid region of western North America^16^. Over 6.8 million ha of the North American Great Basin were dominated by downy brome by 1992, with another 25 million ha where downy brome was a component species^17,18^. In a 2017 mid-season survey of summer-annual crops in Alberta, Canada, annual brome species [including downy brome and Japanese brome (*Bromus japonicas* Houtt.)] were most abundant in the Fescue Grassland, followed by the Moist Mixed Grassland and Mixed Grassland ecoregions^19^. Among summer-annual crops, these annual brome species were most abundant in durum (*Triticum durum* Desf.), followed by barley (*Hordeum vulgare* L.), oat (*Avena sativa* L.), lentil (*Lens culinaris* Medik.), canola, and spring wheat (*Triticum aestivum* L.). Rapid spread of downy brome in southwestern Saskatchewan, Canada, was attributed to increased production of winter cereals grown using conservation tillage practices due the synchrony of the crop and weed life cycles and to the limited number of herbicide options for selective control of downy brome in-crop^20^.

Downy brome is a problematic weed particularly in winter wheat grown in the semi-arid region of western Canada and the United States (US). Infestations of downy brome can increase over-winter mortality of winter wheat presumably by harboring diseases such as snow mold^21^. Downy brome reduced winter wheat yield in Washington by 28% when it was present at a modest density of 54 plants m^-2^ ^22^, while downy brome in Kansas and Wyoming resulted in 10, 15, and 20% winter wheat yield loss when present at densities of 24, 50, and 65 plants m^-2^, respectively^23^. High densities of downy brome up to 538 plants m^-2^ reduced winter wheat yield in Washington by 92%^22^, while in Alberta, winter wheat yield was reduced by 68% when downy brome was present at 400 plants m^−2^^24^. However, the timing of downy brome emergence is a critical factor that governs competition with winter wheat more than downy brome density^24^; suggesting an important role for residual pre-emergence herbicides in delaying the downy brome emergence window^25,26^. For example, winter wheat yield losses were two- to five-fold lower when downy brome emerged six weeks after winter wheat or in the early spring compared with emergence up to three weeks after winter wheat^24^. Similar observations were made in Kansas and Wyoming, where winter wheat yield remained unaffected when downy brome emerged 21 or more days following winter wheat^23^. Fall-emerging downy brome plants can impact summer-annual crops like spring wheat or canola to a greater extent if they remain uncontrolled prior to planting because the downy brome root system continues to grow over winter and in the spring it can deplete moisture in the upper soil layers that would otherwise be available to germinating crop seed^16,27^. Downy brome also creates significant risk of wildfires in rangeland and naturalized areas due to its ability to invade these areas and transform plant communities into a dense downy brome monoculture, combined with the low water content and high flammability of the senesced plant tissue^28^.

Downy brome plants reproduce by seed, and can produce up to 2.6 billion seeds ha^−1^ ^16^. Plants in a dense stand produce around 10–25 seeds plant^−1^, but this ranges up to 6000 seeds plant^−1^ in the absence of competition^16,29,30^. Downy brome plants mature in late-May to early-June, and their seeds shatter within one week after maturity^29^. The seeds are dispersed nearby the parent plant, are moved along the soil surface by wind, through ectozoochory^15,17,29,31,32^, and likely also by farm equipment contamination. Most (96–99%) of the seeds germinate within one year following dispersal^33^. The seed has relatively short longevity in the soil seedbank (2–5 yr)^31,34,35^, with only about 2% of downy brome seeds remaining viable after 3 years^36^. Therefore, limitation of seed return to the soil seedbank can be a critical strategy for long-term management of downy brome populations^37^.

**Figure 1:**
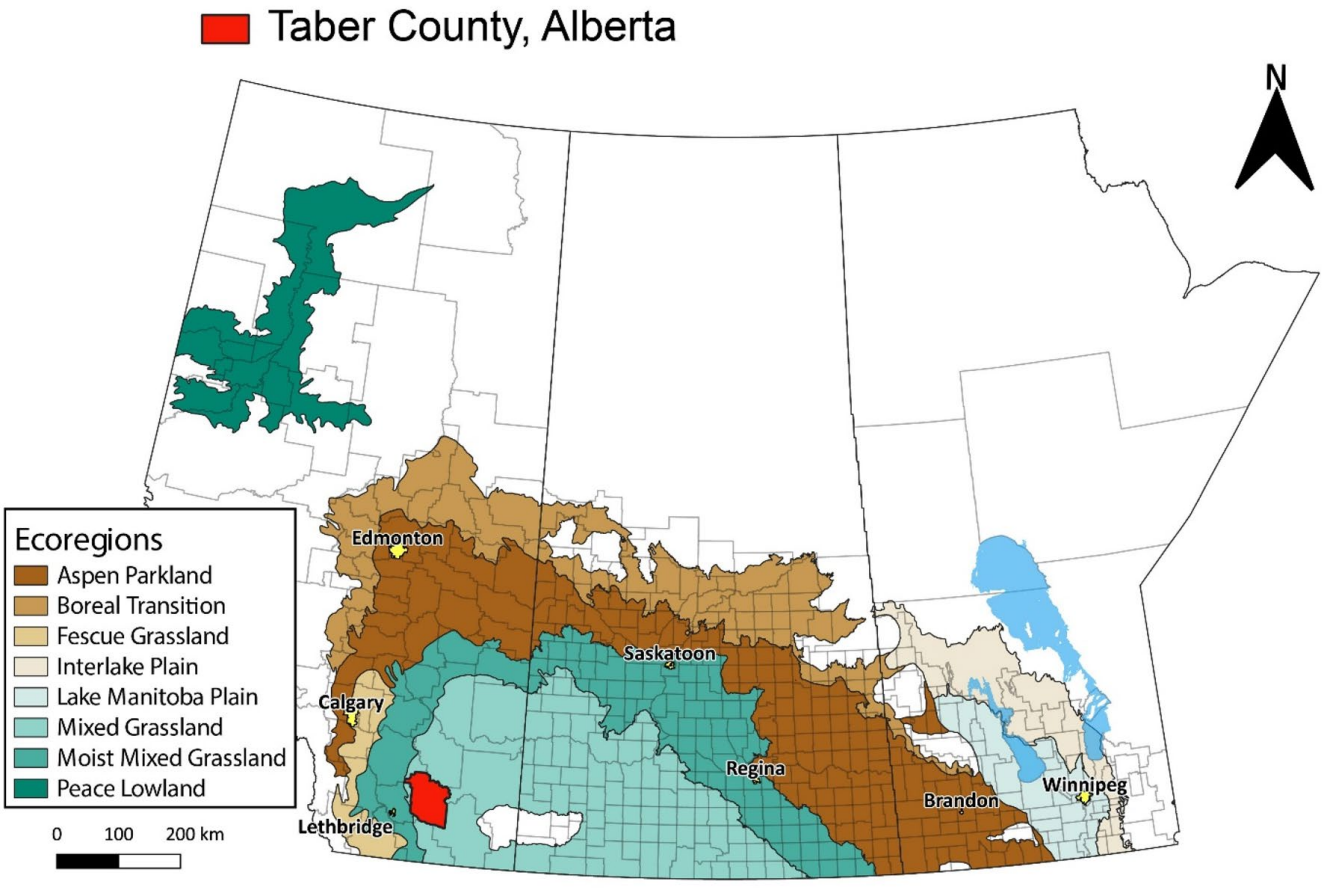
Map of the Canadian prairie region showing the location of Taber County, Alberta, where the first case of glyphosate-resistant downy brome in Canada was confirmed. Developed using QGIS 3.16 (https://qgis.org/en/site/)^56^.

In 2021, lack of control of a downy brome population was observed in a glyphosate-resistant canola field located in Taber County, Alberta, Canada (Fig. 1), following four applications of glyphosate alone (Table 1). Poor herbicidal control can be due to several factors, including misapplication (improper rate, equipment, etc.), poor water quality, inappropriate plant staging, subsequent flushes of emergence, weather during or shortly after application, or the evolution of herbicide resistance. While herbicide-resistant downy brome is not known to occur in Canada^5^, glyphosate resistance was confirmed in three downy brome populations in Washington US prior to 2020^38,39^. The objectives of this research were to determine (a) whether the putative glyphosate-resistant downy brome population collected from Taber County, Alberta was glyphosate-resistant, and if so, (b) the incidence of glyphosate-resistant individuals within the population, and (c) the level of glyphosate resistance exhibited by the population.

**Table 1:** Recent crop and herbicide use history in the field where glyphosate-resistant downy brome was initially confirmed.

Application timing				Crop	Common name	Trade name	Rate g ae/ai ha^-1^	Company^b^
Year	Month	Day	Window^a^					
2020			PP	Spring wheat	Glyphosate	Stonewall® 540	667	WinField® United Canada
2020			POST	Spring wheat	Pyroxsulam	Simplicity™ GoDRI™	70	Corteva Agriscience Canada
2021	April	09	PP	Spring canola	Glyphosate	Stonewall® 540	1134	WinField® United Canada
2021	April	29	PP	Spring canola	Glyphosate	Stonewall® 540	1000	WinField® United Canada
2021	June	01	POST	Spring canola	Glyphosate	Roundup Transorb® HC	934	Bayer CropScience Inc.
2021	June	23	POST	Spring canola	Glyphosate	Roundup Transorb® HC	934	Bayer CropScience Inc.

^a^Application window abbreviations: PP, pre-plant; POST, post-emergence.

^b^Bayer CropScience Inc., Calgary, AB www.cropscience.bayer.ca; Corteva Agriscience Canada Company, Calgary, AB www.corteva.ca; WinField® United Canada, ULC, Saskatoon, SK www.winfieldunited.ca.

## Results

### Glyphosate single-dose population screening

The typical field rate of glyphosate in this region (900 g ae ha^−1^) resulted in complete differentiation of the putative resistant population from both susceptible populations 21 days after treatment (DAT). Among replicates and experimental runs, 100% of the downy brome plants from the putative glyphosate-resistant population exhibited either no injury or some injury with new regrowth 21 DAT, while all plants from both susceptible populations were either dead or nearly dead (data not shown).

### Glyphosate dose-response

**Figure 2:**
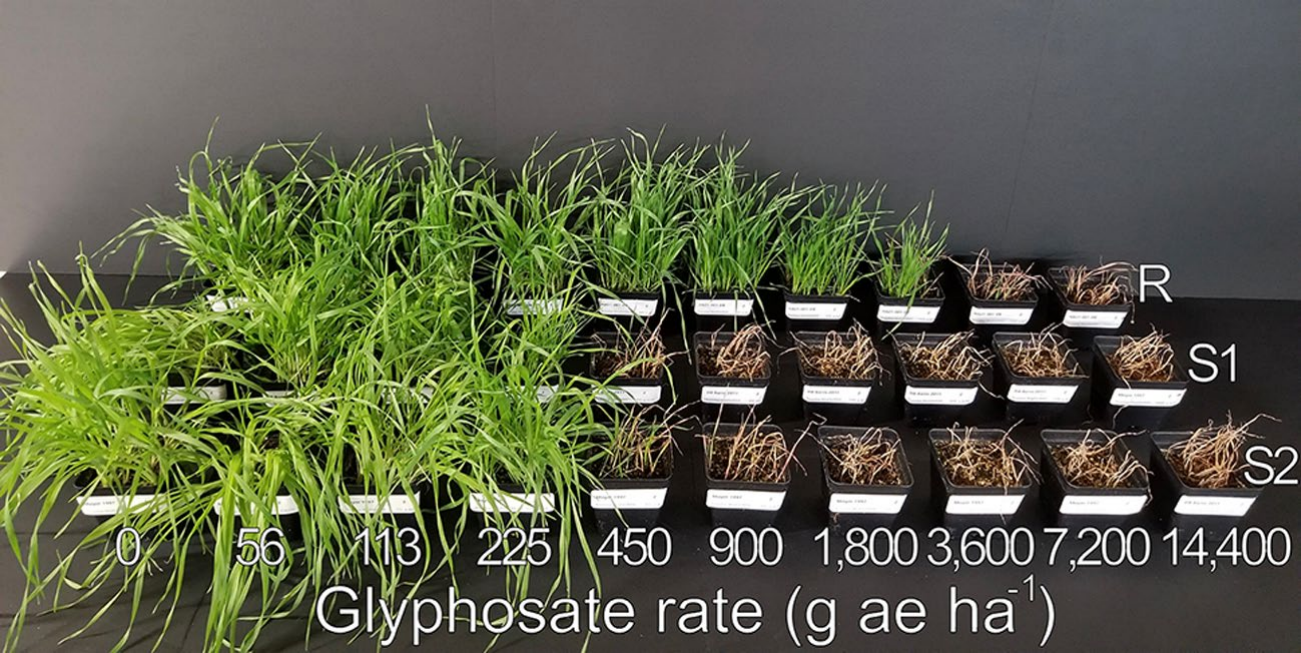
One replicate of the dose-response experiment showing the response of populations Resistant (R; back), Susceptible-1 (S1; middle), and Susceptible-2 (S2; front) to ten rates of glyphosate. The typical field rate of glyphosate in this region is 900 g ae ha^-1^.

**Figure 3:**
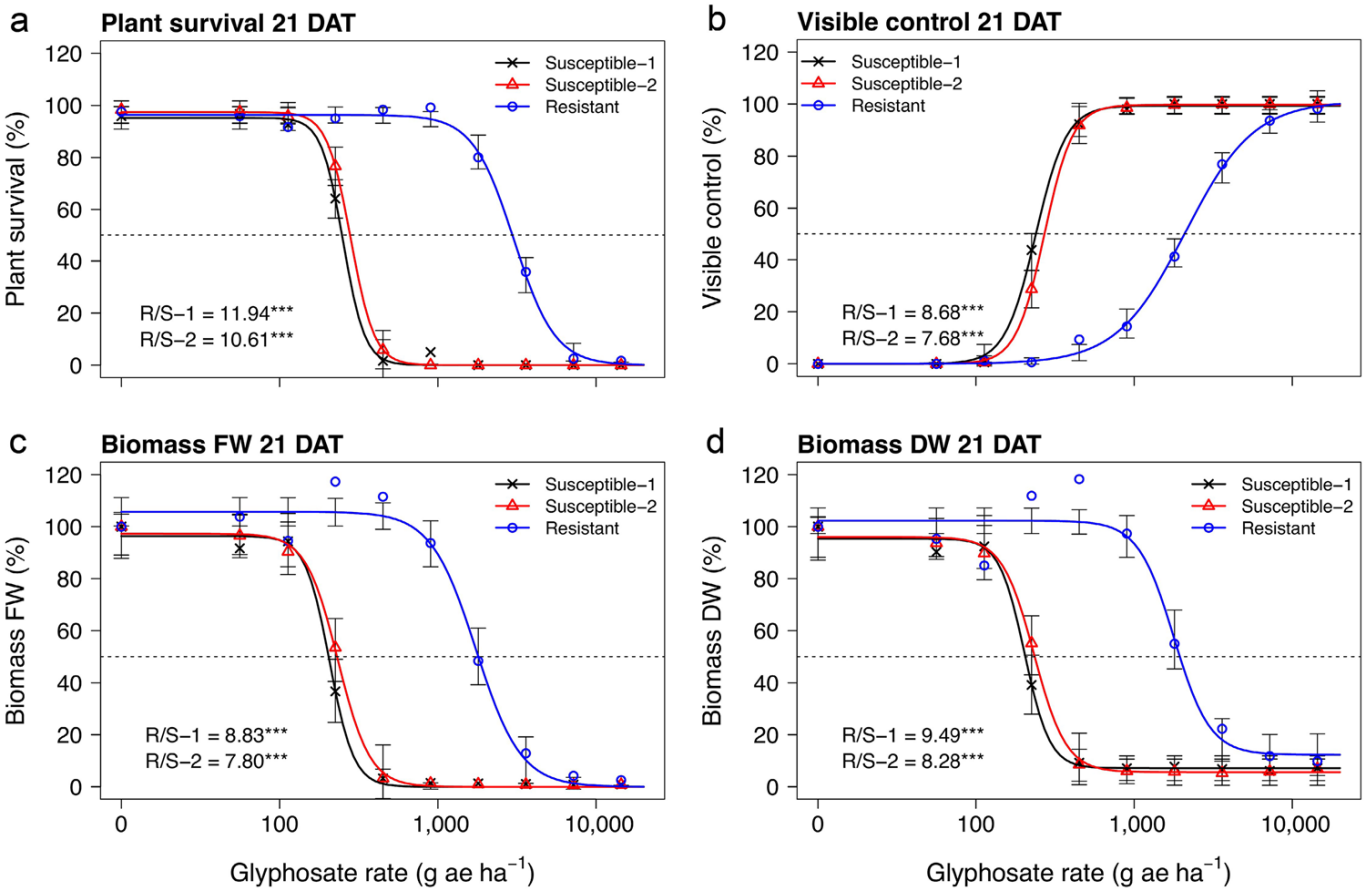
Plant survival (**a**), visible control (**b**), and biomass fresh weight (FW) (**c**) and dry weight (DW) (**d**) of three downy brome populations (Susceptible-1, Susceptible-2, and Resistant) 21 days after treatment (DAT) with ten rates of glyphosate in a combined analysis among experimental runs. Dots indicate means while error bars indicate ± SE. The dashed line indicates the 50% response level and R/S values indicate the resistance indices for the resistant population relative to each susceptible population. *** indicates significant difference in R/S from unity at *P* < 0.001.

**Figure 4:**
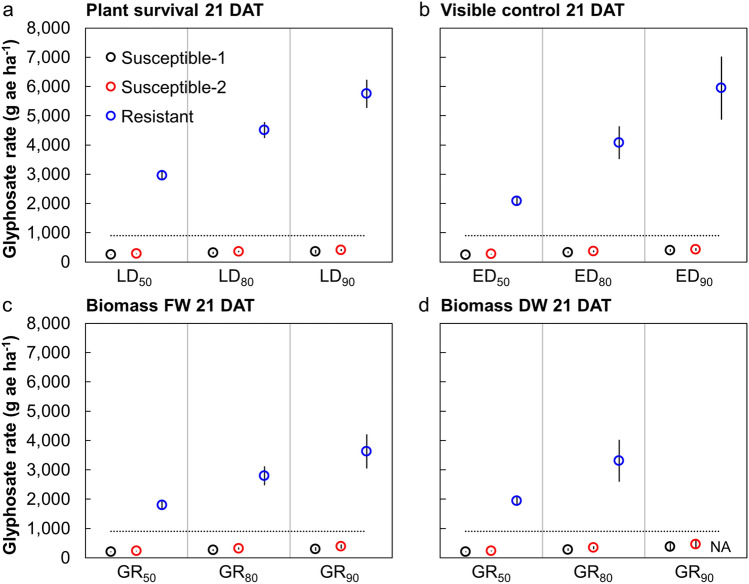
The estimated glyphosate rate required to cause 50%, 80%, and 90% downy brome plant mortality (LD_50_; LD_80_; LD_90_) (**a**), visible control (ED_50_; ED_80_; ED_90_) (**b**), and reduction in biomass fresh weight (FW) (GR_50_; GR_80_; GR_90_) (**c**) and dry weight (DW) (GR_50_; GR_80_; GR_90_) (**d**) 21 days after treatment (DAT). Circles indicate means for each population while bars indicate ± SE. The horizontal dotted line indicates the typical field rate of glyphosate (900 g ae ha^−1^) in this region. NA indicates inestimable values beyond the model asymptotes.

The putative glyphosate-resistant downy brome population exhibited 10.6- to 11.9-fold, 7.7- to 8.7-fold, 7.8- to 8.8-fold, and 8.3- to 9.5-fold resistance (resistance indices) to glyphosate based on plant survival, visible control, and biomass fresh weight (FW) and dry weight (DW), respectively, compared with the two susceptible populations 21 DAT (Figs. 2, 3). The estimated glyphosate rates causing 50% plant mortality (LD_50_), visible control (ED_50_), and reduction in biomass FW and DW (GR_50_) for the susceptible populations ranged from 204 to 279 g ae ha^−1^, while the same rates for the putative glyphosate-resistant population ranged from 1802 to 2961 g ae ha^−1^ (Table 2, Figs. 3, 4). At the 80% response level considered control by herbicide regulators, effective rates for control of the susceptible populations ranged from 262 to 361 g ae ha^-1^, while a similar response from the putative resistant population required glyphosate rates ranging from 2795 to 4511 g ae ha^−1^. The typical field rate of glyphosate in this region (900 g ae ha^−1^) resulted in about 1% (± 0.8 SE) plant mortality, 14% (± 5.4) visible control, and reduction in biomass FW and DW by 6% (± 8.7) and 3% (± 8.0), respectively, for the putative glyphosate-resistant population (data not shown). Therefore, negligible control of the putative glyphosate-resistant downy brome was observed when glyphosate was applied at typical use rates for this region.

**Table 2:** Regression parameter estimates for the four-parameter log-logistic model fit to describe plant survival, visible control, biomass fresh weight (FW), and biomass dry weight (DW) of three downy brome populations 21 days after treatment with a range of glyphosate doses.

Response variable	Population	*b* (± SE)	*c* (± SE)^a^	*d* (± SE)	*e* (± SE)
**Survival**					
*RSE* = 10.6	Susceptible-1	6.3 (2.4)		95.2 (2.2)	252 (12)
	Susceptible-2	5.8 (1.0)		97.4 (2.2)	281 (13)
	Resistant	3.4 (0.4)		96.3 (1.6)	3029 (142)
**Visible control**					
*RSE* = 10.4	Susceptible-1	-4.5 (0.9)		99.4 (1.7)	239 (9)
	Susceptible-2	-4.9 (0.8)		99.7 (1.7)	271 (11)
	Resistant	-2.0 (0.3)		101.2 (3.9)	2106 (159)
**Biomass FW**					
*RSE* = 17.4	Susceptible-1	5.7 (3.6)		96.3 (4.3)	207 (15)
	Susceptible-2	4.3 (1.6)		97.3 (4.2)	234 (15)
	Resistant	3.1 (0.6)		105.7 (2.8)	1740 (123)
**Biomass DW**					
*RSE* = 16.3	Susceptible-1	5.5 (3.3)	7.1 (2.4)	95.4 (4.2)	203 (16)
	Susceptible-2	4.3 (1.7)	5.6 (2.6)	96.0 (3.9)	233 (15)
	Resistant	3.8 (1.1)	12.3 (4.1)	102.3 (2.5)	1787 (137)

*b* slope of the response curve at the inflection point, *c* lower asymptote, *d* upper asymptote, *e* response curve inflection point, *RSE* residual standard error.

^a^When the lower asymptote ‘*c*’ did not differ from zero, the model collapsed to a three-parameter log-logistic model where the lower asymptote was fixed at zero.

## Discussion

Results from the current study confirm the presence of glyphosate-resistant downy brome in a glyphosate-resistant canola field in Taber County, Alberta, in 2021 (Fig. 1). While glyphosate resistance has been reported in six broadleaf weed species in Canada since 2010^5^, this study represents the first confirmation of a glyphosate-resistant grass weed in Canada. It also represents the second glyphosate-resistant weed reported in the prairie region of Canada (region shown in Fig. 1), which consists of 84% of the 47.8 million ha arable land base in the country^9^.

Confirmation of glyphosate-resistant downy brome in Canada follows previous reports of glyphosate resistance in poverty brome (*Bromus sterilis* L.) in central England (2010)^40^, ripgut brome (*Bromus diandrus* Roth) in Australia (2011)^41^, red brome (*Bromus rubens* L.) in southern Spain (2018)^42^, and downy brome in Washingon, US (prior to 2020)^38,39^. The three glyphosate-resistant downy brome populations from Washington, US exhibited 88- to 165-fold resistance to glyphosate compared with a susceptible biotype. While the level of glyphosate resistance exhibited by the Washington populations was greater than the Alberta population in the current study (Figs. 2, 3, 4), it is important to note that the glyphosate resistance indices ranging from 7.7 to 11.9 in the current study were well above the threshold of 4 for confirmation of herbicide resistance^5^. The three glyphosate-resistant downy brome populations confirmed in Washington were not resistant to other herbicide sites of action^39^. However, among 50 downy brome populations tested in Washington between 2013 and 2020, 2% were resistant to both acetyl-CoA carboxylase (ACCase) inhibitors (HRAC Group 1) and acetolactate synthase (ALS) inhibitors (HRAC Group 2), 52% were cross-resistant to multiple chemical families of ALS inhibitors, and 20% were resistant to a single chemical family of ALS-inhibiting herbicides. The overall occurrence of resistance in downy brome populations from Washington varied among herbicide active ingredients, and was greatest for imazamox (66% of the populations tested), followed by propoxycarbazone (53%), mesosulfuron (47%), sulfosulfuron (46%), pyroxsulam (35%), glyphosate (6%), and clethodim (2%). Elsewhere, ACCase inhibitor-resistant downy brome was documented in Oregon, US in 2005, while populations with resistance to ALS-inhibiting herbicides were documented in Oregon and Montana, US in 1997 and 2015, respectively^5,43,44^. Photosystem II inhibitor (HRAC Group 5)-resistant downy brome was documented first in France and Spain in 1981 and 1990, respectively, but is not known to occur elsewhere^5^. In contrast, herbicide resistance was not known to occur in downy brome populations in Canada prior to the current study. Future research will assess potential cross- or multiple-resistance to other herbicide sites of action in this glyphosate-resistant downy brome population. Mitigating the evolution and spread of downy brome with resistance to ACCase- or ALS-inhibiting herbicides will be critical to preserving the remaining effective post-emergence herbicide options for management of glyphosate-resistant downy brome in western Canada and the US.

Understanding the mechanism of glyphosate resistance in the Alberta downy brome population may provide insight into the risk of spread beyond this initial confirmation and enhanced mitigation measures. Mutations in the 5-enolpyruvylshikimate-3-phosphate synthase (*EPSPS*) gene known to confer glyphosate resistance in other species^45^ were not observed in the three glyphosate-resistant downy brome populations from Washington^38^. However, *EPSPS* gene copy number was 14- to 18-fold greater in the glyphosate-resistant populations compared with the -susceptible population, resulting in increased *EPSPS* expression by about 7.5- to 9-times; even though no correlation was observed among *EPSPS* copy number and expression levels in the resistant populations. Thus, the likely mechanism conferring glyphosate resistance in downy brome is *EPSPS* gene amplification, similar to the mechanism conferring glyphosate resistance in other economically damaging weed species like kochia [*Kochia scoparia* (L.) Schrad.]^46^ and Palmer amaranth (*Amaranthus palmeri* S. Wats.)^47^ in addition to another brome species ripgut brome (*Bromus diandus* Roth)^41^. Future research should confirm whether this mechanism is indeed that which confers glyphosate resistance in the Alberta downy brome population.

Further surveillance efforts are required to fully understand the scope and impact of glyphosate-resistant downy brome in western Canada and the US. Enhanced monitoring of downy brome populations will comprise a critical mitigation measure to limit the spread of glyphosate-resistant downy brome in Alberta as well as neighboring provinces and states. While effective pre- and post-emergence herbicide options^25,26,48,49^ will play an important role in managing glyphosate-resistant downy brome, integration of non-chemical management strategies will be necessary to mitigate selection for resistance to these remaining herbicide options. Growing competitive cultivars^50^, crop rotations involving summer-annuals^51,52^, strategic timing of nitrogen fertilization^53^, and judicious use of tillage^54^ may help reduce selection pressure for herbicide-resistant downy brome in cropping systems. Spread of glyphosate-resistant downy brome beyond the field where it was originally confirmed is seed-limited due to minimal cross-pollination in this species^39,55^. Therefore, cleaning of equipment and seed products, and minimizing viable downy brome seeds in livestock feed, will be critical to limit the spread of glyphosate-resistant downy brome among farms and fields.

## Methods

### Plant material and recent management history

Mature seed was collected from the putative glyphosate-resistant downy brome population in early July 2021. About 100 downy brome plants were sampled at random from the glyphosate-resistant canola field in Taber County, Alberta (location reported at the county scale to protect farmer identity), and seeds from all plants were combined into a single sample representing the downy brome population (referred to as “Resistant”). The collected seeds were cleaned by hand, homogenized by mixing, dried at ambient room temperature, then stored at 4 °C until use. A similar protocol was used to collect seeds from two known susceptible populations located near Lethbridge, Alberta (referred to as “Susceptible-1” and “Susceptible-2”). A questionnaire was used to collect information on recent management history from the field where the putative glyphosate-resistant downy brome population was collected, including the crops grown and herbicides applied over the past two years (Table 1). The location of Taber County, where the putative glyphosate-resistant downy brome population was collected (Fig. 1), was mapped using QGIS 3.16^56^. All plant materials were handled according to relevant institutional, provincial and federal guidelines and regulations. Land access permission was granted by the land owner following the procedures of Agriculture and Agri-Food Canada.

### Glyphosate single-dose bioassays

Single-dose whole-plant bioassays were used in an initial screening step to determine the incidence of glyphosate-resistant individuals within the populations. The experiment was designed as a two-way factorial randomized complete block design (RCBD) with three populations (Resistant, Susceptible-1, and Susceptible-2) and two herbicide treatments (treated and untreated). The experiment included three replicates and was repeated over two runs. Seeds from each of the three populations were planted in separate 52 × 26 × 5 cm greenhouse flats, each containing an insert with 72 equally-sized compartments. Each compartment was filled with modified Cornell soilless potting mixture^57^ containing 756 mg N, 958 mg P, and 505 mg K L^−1^ mixture. The downy brome seeds were planted at 1 cm depth, and emerged seedlings were thinned to a single plant in each of the 72 compartments per flat. The flats were placed in the greenhouse under 20/18 °C day/night temperature, with a 16 h photoperiod supplemented with 100 µmol m^-2^ s^-1^ light, and watered daily.

When the downy brome plants reached the 2-leaf stage, they were treated with glyphosate (Roundup WeatherMax®, Bayer CropScience, Calgary, AB) at 900 g ae ha^−1^ using a moving-nozzle cabinet sprayer. The sprayer was equipped with a flat-fan 8002VS TeeJet® nozzle (Spraying Systems Co., Wheaton, IL), and calibrated to deliver 200 L ha^−1^ spray solution at 275 kPa and a speed of 2.4 km hr^-1^. The flats were returned to the greenhouse following treatment.

Treated plants were rated individually as either resistant (no injury, or some injury with new regrowth) or susceptible (dead, or nearly dead) 21 DAT by comparing with the corresponding untreated flats for each population. The incidence of glyphosate-resistant individuals was determined as a percentage of the total number of individuals treated (72) within each flat.

### Glyphosate dose-response bioassays

Dose-response whole-plant bioassays were used to determine the level of glyphosate resistance exhibited by the putative glyphosate-resistant downy brome population by comparing with two known susceptible populations. The experiment was designed as a two-way factorial RCBD with three populations (Resistant, Susceptible-1, and Susceptible-2) and 10 glyphosate rates (0, 56, 113, 225, 450, 900, 1800, 3600, 7200, and 14400 g ae ha^−1^) equivalent to 0, 0.063, 0.125, 0.25, 0.5, 1, 2, 4, 8, and 16 times the typical glyphosate field rate in this region. The experiment included four replicates and was repeated over two runs. Seeds from each population were planted at 1 cm depth in 10 × 10 × 12 cm plastic greenhouse pots filled with the soilless potting mixture described above. The pots were placed in the greenhouse under the aforementioned conditions and watered daily. Emerged seedlings were thinned to 15 plants pot^−1^. The herbicide treatments were applied when the downy brome reached the 2-leaf stage using the moving-nozzle cabinet sprayer described above. The pots were returned to the greenhouse following treatment.

Downy brome plant survival, visible control, and shoot biomass FW and DW were determined 21 DAT. Plant survival was determined by rating each individual plant as living (no injury, or some injury with new regrowth) or dead (dead, or nearly dead), and expressing the number of living plants as a percentage of the total number of plants treated (15) within each pot. Visible control was assessed as a percentage from 0% (no injury) to 100% (complete necrosis) relative to the untreated control for each population following the herbicide efficacy ratings described by CWSS-SCM^58^. All aboveground shoot tissue was clipped at the soil surface, and shoot FW determined. The shoot tissue was dried at 60 °C until equilibrium, and shoot DW determined.

### Statistical analyses

No variation was observed among replicates and runs of the single-dose whole-plant bioassays. Therefore, resistance incidence data did not conform to the assumptions of analysis of variance (ANOVA), and simple means are presented.

Plant survival, visible control, and shoot biomass FW and DW data from the dose-response bioassays were subjected to ANOVA using the MIXED procedure of SAS Studio 3.81 (SAS Institute Inc., Cary, NC). Population, glyphosate rate, experimental run, and their interactions were considered fixed effects, while replication nested within experimental run was considered a random effect. The UNIVARIATE procedure was used to assess the assumption of normality using the Shapiro–Wilk test, while visual inspection of the residuals and predicted values was used to assess the homogeneity of variance^59^. Variance component analyses using type III sums of squares showed that all main and interaction effects including experimental run accounted for < 5% of the model sums of squares, and therefore subsequent analyses were combined across runs.

The dose-response data were subjected to nonlinear regression using the ‘drc’ package in R v. 3.6.0^60^. Each response variable comprised a separate analysis, and a separate dose-response curve was fit for each population using the four-parameter log-logistic Eq. (1):

1 $$y=c+\left\{d-c/1+\mathrm{exp}\left[b\left(\mathrm{log} \, x-\mathrm{log} \, e\right)\right]\right\}$$

where: $$y$$ is the response variable, $$c$$ is the lower asymptote, $$d$$ is the upper asymptote, $$b$$ is the slope of the regression curve at dose $$e$$, $$e$$ is the regression curve inflection point, and $$x$$ is the glyphosate rate^61,62^. In cases where the lower asymptote $$c$$ did not differ from zero (α = 0.05) (for plant survival, visible control, and biomass FW), a common value was fit ($$c=0)$$, and the log-logistic model collapsed to its three-parameter form. The fit of each nonlinear model was assessed using the lack-of-fit test (*P* > 0.05). The glyphosate rates causing 50, 80, and 90% plant mortality (LD_50_, LD_80_, and LD_90_, respectively), visible control (ED_50_, ED_80_, and ED_90_, respectively), and reduction in biomass FW and DW (GR_50_, GR_80_, and GR_90_, respectively) were extracted for each population. The ‘EDcomp’ function was used estimate the resistance index by comparing the glyphosate rate at the 50% response level for the resistant population with that of each susceptible population (α = 0.05)^61^. The putative glyphosate-resistant downy brome population was considered resistant if the resistance indices were ≥ 4^5^.

## Data Availability

Data for this study will be made available by request to the corresponding author.
